# Biosensing Platforms and Multimodal Monitoring of Sepsis-Associated Cell Death

**DOI:** 10.3390/bios16100531

**Published:** 2026-09-23

**Authors:** Oana Coman, Leonard Azamfirei, Oana Frandeș, Bianca-Liana Grigorescu

**Affiliations:** 1 Department of Simulation Applied in Medicine, George Emil Palade University of Medicine, Pharmacy, Science, and Technology of Târgu Mureș, 540139 Târgu Mureș, Romania; oana.coman@umfst.ro; 2 Department of Anaesthesiology and Intensive Care, George Emil Palade University of Medicine, Pharmacy, Science, and Technology of Târgu Mureș, 540139 Târgu Mureș, Romania; bianca.grigorescu@umfst.ro; 3 Doctoral School of Medicine and Pharmacy, George Emil Palade University of Medicine, Pharmacy, Science, and Technology of Târgu Mureș, 540139 Târgu Mureș, Romania; oana.frandes@umfst.ro

**Keywords:** apoptosis, sepsis, immune dysfunction, biomarkers, biosensors, artificial intelligence, machine learning, multimodal monitoring, cell death, longitudinal monitoring

## Abstract

Apoptosis plays an important role in sepsis-associated immune dysfunction, particularly through lymphocyte depletion and the development of persistent immunosuppression. This review examines apoptosis-associated biomarkers in sepsis, distinguishing relatively direct markers of cell death from complementary indicators of immune dysfunction, inflammation, and organ injury, and considers their potential for longitudinal monitoring. Particular attention is given to biosensing platforms that may enable rapid, low-volume, and multiplexed detection of these biomarkers. Representative electrochemical, optical, nanoparticle-assisted, and microfluidic approaches are discussed in terms of their analytical performance, biological relevance, and stage of clinical translation. The review also considers how serial biosensor measurements could be combined with physiological signals, conventional laboratory parameters, and organ dysfunction scores to follow changes in the patient’s biological and clinical status over time. Artificial intelligence and machine-learning methods may facilitate the interpretation of these complex datasets and support multimodal patient assessment. However, most biosensors targeting relatively direct apoptosis-associated markers remain at the experimental stage, and their added clinical value in sepsis has not yet been established. Future studies should therefore determine whether serial assessment of cell-death-associated biomarkers provides meaningful information beyond currently available inflammatory biomarkers, physiological monitoring, and severity scores.

## 1. Introduction into Sepsis Pathophysiology

Sepsis is a life-threatening organ dysfunction caused by a dysregulated host response to infection and remains a major cause of morbidity and mortality worldwide [1,2]. Its pathophysiology involves a complex interaction between inflammation, immune dysfunction, endothelial and microcirculatory injury, mitochondrial dysfunction, and coagulation abnormalities [2,3]. Pro-inflammatory and immunosuppressive responses may occur sequentially or coexist during the course of the disease, contributing to substantial biological heterogeneity between patients [2,4,5]. Conventional physiological variables, laboratory parameters, and organ dysfunction scores are essential for clinical assessment but provide limited information on the molecular mechanisms underlying this heterogeneity; thus, identifying molecular processes that precede overt organ dysfunction has become an important objective in contemporary sepsis research [6,7].

Apoptosis is particularly relevant in this context because excessive immune-cell death contributes to the loss of CD4+ and CD8+ T lymphocytes, B lymphocytes, and natural killer cells, promoting immunosuppression, impaired pathogen clearance, and susceptibility to secondary infections [8,9]. Persistent immune dysfunction has consistently been associated with prolonged hospitalization and poor clinical outcomes [10,11,12]. Other forms of regulated cell death, including pyroptosis, necroptosis, and ferroptosis, also contribute to cellular and organ injury during sepsis and interact with inflammatory signaling pathways [13]. However, given the established role of apoptosis in lymphocyte depletion and sepsis-induced immunosuppression, apoptotic pathways remain particularly relevant for biomarker-based monitoring. 

Direct assessment of apoptosis is not currently part of routine sepsis management. Among the candidate biomarkers, activated caspases, particularly caspase-3 and caspase-8, and phosphatidylserine externalization are of particular interest because they reflect molecular or cellular events occurring during apoptosis. Immune-checkpoint molecules such as Programmed cell death protein-1 (PD-1) and Programmed death ligand-1 (PD-L1), although not specific markers of apoptosis, may provide complementary information on lymphocyte exhaustion and sepsis-induced immunosuppression [7,8,9]. Detecting these changes could help characterize the immune status of patients beyond conventional measures of inflammation and organ dysfunction, while serial measurements may provide information on the evolution of immune dysfunction during the course of sepsis. Their clinical role, however, remains investigational, and further validation is required before they can be incorporated into routine monitoring.

The purpose of this narrative review is to analyze apoptosis and related regulated cell death pathways as biologically relevant processes in sepsis and to discuss how cell-death-associated biomarkers, physiological signals, and biosensing technologies can support integrative assessment, risk stratification, and the transition toward personalized intensive care. Importantly, the biomarkers and physiological variables discussed in this review differ in their relationship to cell death. Some, such as activated caspases and phosphatidylserine externalization, directly reflect molecular or cellular events associated with regulated cell death, whereas others provide indirect information on immune dysfunction, inflammation, tissue injury, or organ dysfunction. These indirect parameters are included not as specific markers of cell death, but as complementary clinical signals that may provide contextual information when integrated with more specific molecular and cellular biomarkers.

The distinctive perspective of this review lies in considering these elements not as separate areas of sepsis research, but as components of a longitudinal monitoring strategy. Rather than discussing cell-death biology, biosensing technologies, physiological monitoring, and artificial intelligence independently, we examine how changes in apoptosis-associated signals over time could be captured by biosensing platforms and interpreted alongside longitudinal physiological and clinical data. A central question is whether these measurements can provide additional information beyond that already available from conventional inflammatory biomarkers, physiological variables, and organ dysfunction scores. On this basis, we identify current translational gaps and propose priorities for the future validation of apoptosis-informed multimodal monitoring in sepsis.

## 2. Apoptosis and Regulated Cell Death in Sepsis

### 2.1. Apoptosis and Related Cell-Death Pathways

Sepsis is characterized by profound changes in the host immune response, with pro-inflammatory and immunosuppressive mechanisms often occurring simultaneously. Early release of mediators such as Tumor necrosis factor (TNF)-α, interleukin (IL)-1β, IL-6, IL-12, and IL-18 is accompanied by progressive immune dysfunction, which may ultimately lead to persistent immunosuppression [14,15]. A major component of this process is the apoptotic loss of immune cells, particularly CD4+ and CD8+ T lymphocytes, B lymphocytes, natural killer cells, and follicular dendritic cells [16,17]. Lymphocyte depletion compromises both innate and adaptive immune responses, while monocytes and macrophages may acquire a tolerant phenotype characterized by reduced pro-inflammatory cytokine production and altered gene expression. Together, these changes contribute to the immunosuppressive state observed in patients with sepsis [12,18].

The PD-1/ PD-L1 axis is closely involved in this immune dysfunction. Under physiological conditions, PD-1 signaling limits excessive activation of the adaptive immune response and contributes to immune homeostasis. During sepsis, sustained expression of PD-1 and PD-L1 suppresses T-cell receptor signaling, reducing lymphocyte proliferation and effector function and promoting immune exhaustion and apoptosis [19]. PD-L1 is expressed by several immune-cell populations, including T and B lymphocytes and antigen-presenting cells, as well as by non-lymphoid tissues [19]. Persistent activation of this pathway has been associated with secondary infections, multiple organ dysfunction, and poor clinical outcomes and has therefore attracted interest as a potential target for immunomodulatory therapy [10,20].

Apoptosis proceeds mainly through two interconnected pathways: the intrinsic, or mitochondrial, pathway and the extrinsic, or death receptor, pathway. The intrinsic pathway is activated by cellular stressors such as oxidative stress, Deoxyribonucleic acid (DNA) damage, and mitochondrial dysfunction and is regulated by members of the Bcl-2 protein family [21,22]. Activation and oligomerization of the pro-apoptotic proteins Bax and Bak increase mitochondrial outer membrane permeability, allowing cytochrome c to enter the cytoplasm. This promotes apoptosome formation and activation of caspase-9, followed by downstream executioner caspases, particularly caspase-3 [23]. The extrinsic pathway begins with activation of cell-surface death receptors, including Fas, Tumor necrosis factor receptor 1 (TNFR1), and Tumor necrosis factor-related apoptosis-inducing ligand (TRAIL) receptors. Ligand binding initiates signaling complexes that can activate caspase-8, which in turn activates executioner caspases directly or amplifies apoptosis through interaction with the mitochondrial pathway [24]. Both pathways ultimately converge on executioner caspases, resulting in the controlled dismantling and clearance of the affected cell. Excessive immune-cell apoptosis in sepsis contributes to lymphocyte depletion, impaired pathogen clearance, immune paralysis, and adverse clinical outcomes [25].

Apoptosis does not occur in isolation, and other forms of regulated cell death contribute to the inflammatory and tissue responses observed during sepsis. Pyroptosis is a pro-inflammatory form of cell death characterized by gasdermin-mediated membrane pore formation, cellular swelling, membrane rupture, and release of intracellular contents. The canonical pathway is commonly initiated by inflammasome activation, particularly the NOD-like receptor family pyrin domain-containing 3 (NLRP3) inflammasome, in response to pathogen-associated and damage-associated molecular patterns. Recruitment of apoptosis-associated speck-like protein containing a caspase recruitment domain (ASC) and pro-caspase-1 leads to caspase-1 activation, cleavage of gasdermin D (GSDMD), and maturation of IL-1β and IL-18. The N-terminal fragment of GSDMD forms membrane pores that facilitate cytokine release and eventually lead to loss of membrane integrity [26]. A non-canonical pathway mediated by caspase-4 and caspase-5 in humans can also activate GSDMD in response to intracellular lipopolysaccharide and may amplify the canonical pathway through secondary NLRP3 activation [13,26].

The effects of pyroptosis depend on its extent and cellular context. While a controlled response may contribute to elimination of infected cells, excessive pyroptotic activity can amplify systemic inflammation and tissue injury. The NLRP3–caspase-1–GSDMD axis has consequently been implicated in dysfunction of several organs during sepsis, and its components have been investigated as potential indicators of inflammatory cell death. However, their clinical value as diagnostic or prognostic biomarkers remains insufficiently established [26,27].

TNF-α provides an additional link between inflammation and regulated cell death. Binding of TNF-α to TNFR1 can produce different cellular responses depending on the signaling context. Under conditions favoring death signaling, formation of a cytosolic complex involving Receptor-Interacting Serine/Threonine-Protein Kinase (RIPK)1, Tumor necrosis factor receptor type 1-associated death domain protein (TRADD), Fas-associated death domain (FADD), and caspase-8 promotes activation of downstream executioner caspases and apoptosis. When caspase-8 activity is inhibited, signaling may instead shift toward RIPK1/RIPK3/ Mixed lineage kinase domain-like protein (MLKL)-dependent necroptosis, illustrating the close interaction between apoptotic and necroptotic pathways [28].

TNF-α also contributes to endothelial dysfunction by promoting loss of barrier integrity, glycocalyx injury, increased vascular permeability, leukocyte adhesion, and a procoagulant endothelial phenotype. The resulting abnormalities in microvascular perfusion can impair tissue oxygen delivery and contribute to organ dysfunction even when systemic hemodynamic variables have been restored [29,30]. TNF-α may therefore contribute to sepsis-associated injury both through TNFR1-dependent cell-death signaling and through its effects on the endothelium and microcirculation. These mechanisms form part of a broader interaction between inflammation, immune dysfunction, altered tissue perfusion, mitochondrial injury, and regulated cell death throughout the course of sepsis [31].

### 2.2. Biomarkers and Methods for Cell-Death Assessment

Apoptosis can be assessed at different stages of the cell-death process using molecular biomarkers and cellular assays. Molecular markers provide evidence of activation of specific apoptotic signaling pathways, whereas cellular methods identify structural and biochemical changes occurring as cells progress through apoptosis. These approaches are complementary and can provide information on both the mechanisms involved and the extent of apoptotic cell loss.

Among the molecular markers, caspases are particularly relevant because of their central role in the apoptotic cascade. Caspase-3 is the principal executioner caspase, whereas caspase-8 and caspase-9 are associated predominantly with activation of the extrinsic and intrinsic pathways, respectively. Other markers, including Fas/FasL, cytochrome c release, and changes in the Bcl-2/Bax ratio, provide additional information on the initiation and regulation of programmed cell death. These biomarkers have been investigated in experimental models and clinical studies of sepsis, although their use remains largely confined to research settings [32].

At the cellular level, one of the earliest detectable changes is the externalization of phosphatidylserine (PS) from the inner to the outer leaflet of the plasma membrane. Under physiological conditions, exposed PS acts as a recognition signal for the clearance of apoptotic cells with limited induction of inflammation. In sepsis, increased PS exposure has been described in several cell populations, including endothelial cells, platelets, erythrocytes, and leukocytes, as well as on extracellular vesicles. In addition to indicating apoptotic activity, excessive PS exposure may contribute to inflammatory and coagulation abnormalities, endothelial injury, and microvascular dysfunction [33].

Annexin V, a calcium-dependent phospholipid-binding protein with high affinity for PS, is widely used to identify PS externalization. When combined with a membrane-impermeant viability dye and analyzed by flow cytometry, Annexin V staining can distinguish viable cells from cells with early PS externalization and from cells that have also lost membrane integrity. This approach is therefore widely used to characterize different stages of cell death, although PS externalization should be interpreted in conjunction with membrane integrity and other cellular features rather than as an isolated measure of apoptosis [34].

DNA fragmentation can be assessed using the terminal deoxynucleotidyl transferase dUTP nick-end labeling (TUNEL) assay, which detects DNA strand breaks that occur during later stages of apoptosis. TUNEL therefore provides information complementary to Annexin V-based assessment, particularly when the progression of cell death is being evaluated. However, because DNA fragmentation is not exclusive to apoptosis, TUNEL findings also require interpretation in the appropriate morphological and experimental context [35].

Multiparametric flow cytometry extends cellular assessment beyond the detection of apoptosis alone by allowing apoptotic markers to be analyzed together with immune-cell phenotype. Annexin V and viability staining can be combined with markers identifying CD4+ and CD8+ T lymphocytes, B lymphocytes, natural killer cells, and monocytes, as well as immune-checkpoint molecules such as PD-1 and PD-L1. This makes it possible to determine which immune-cell populations are affected and to examine cell loss alongside changes in activation or exhaustion at the single-cell level. Flow cytometry is therefore an important research tool for characterizing sepsis-associated immune dysfunction, although requirements for fresh samples, specialized instrumentation, standardized protocols, and experienced personnel continue to limit its routine bedside application [3].

From a biosensing perspective, candidate biomarkers differ considerably in their biological specificity, accessibility, and suitability for detection. Activated caspases and phosphatidylserine externalization provide relatively direct information on apoptotic activity, whereas PD-1/PD-L1 reflects the accompanying immune dysfunction and other routinely available biomarkers provide complementary information on inflammation and organ injury. The biological relationship of these markers to apoptosis and their potential suitability for biosensor-based detection are summarized in Figure 1.

### 2.3. Complementary Clinical and High-Dimensional Indicators

Direct assessment of apoptosis is not routinely available in clinical practice; thus, several readily accessible clinical and laboratory parameters have been investigated as indirect indicators of the immune and organ dysfunction that accompanies sepsis-associated cell death. These parameters should not be considered biomarkers of apoptosis or other regulated cell death pathways per se. Rather, they reflect downstream or concomitant processes, including lymphocyte depletion, immune exhaustion, inflammation, tissue injury, and organ dysfunction, and may therefore provide complementary information when interpreted alongside more specific molecular or cellular markers of cell death.

In addition to immune-related biomarkers, routinely available indicators of organ dysfunction provide valuable information regarding the systemic consequences of regulated cell death during sepsis. Although these parameters are not specific for apoptosis or other regulated cell death pathways, they reflect tissue injury occurring in the liver, myocardium, kidneys, and other organs as a result of inflammation, endothelial dysfunction, microcirculatory impairment, mitochondrial dysfunction, and programmed cell death. Representative indirect clinical indicators and their relationship with organ dysfunction are summarized in Table 1 [13,36].

Persistent lymphopenia is one of the most widely studied clinical indicators associated with sepsis-induced immune dysfunction. Sustained reductions in circulating lymphocyte counts are consistent with the profound immune alterations observed during sepsis and have been associated with secondary infections, multiple organ dysfunction, and increased mortality. Because lymphocyte counts are readily obtained from routine complete blood counts, persistent lymphopenia provides a simple and inexpensive means of monitoring immune competence over time. Its relationship with apoptosis, however, is indirect, since reduced circulating lymphocyte counts may also result from redistribution, impaired lymphopoiesis, or other pathological processes [12].

Composite inflammatory indices derived from routine blood counts, including the platelet-to-lymphocyte ratio (PLR) and neutrophil-to-lymphocyte-to-monocyte ratio (NLMR), have also been investigated as indicators of the balance between systemic inflammation and immune dysfunction. Higher values have been associated with disease severity and adverse outcomes in patients with sepsis. These indices are inexpensive and readily available and may therefore be useful for risk stratification and serial monitoring, but they remain nonspecific and should not be interpreted as direct measures of apoptosis [37].

Immune-checkpoint molecules occupy an intermediate position between direct markers of cell death and conventional clinical indicators. In particular, increased PD-1/PD-L1 signaling has been associated with lymphocyte exhaustion, impaired immune function, secondary infections, multiple organ dysfunction, and adverse outcomes in sepsis. Assessment of PD-1 and PD-L1 may therefore provide information on the immunosuppressive phenotype of individual patients and has also attracted interest in the context of immune-checkpoint-directed therapies. Nevertheless, PD-1 and PD-L1 are markers of immune dysfunction rather than specific measures of apoptosis, and their clinical application in sepsis remains investigational [19].

The clinical relevance of these biomarkers and indicators ultimately depends on their association with meaningful patient outcomes. Studies have linked persistent lymphopenia, immune-checkpoint expression, pyroptosis-associated markers, and conventional indicators of organ injury with mortality, secondary infections, multiple organ dysfunction, mechanical ventilation, length of hospitalization, and established severity scores. Representative clinical studies illustrating these associations are summarized in Table 2 [12,38,39,40,41,42].

High-dimensional approaches provide a different perspective by examining multiple components of the host response simultaneously rather than relying on individual biomarkers. Transcriptomic and proteomic analyses have identified gene-expression patterns and protein signatures associated with immune dysfunction, disease severity, organ injury, and clinical outcomes in sepsis. These methods can capture biological heterogeneity that may not be apparent from individual circulating biomarkers, although interpretation of the resulting molecular signatures remains complex [43,44].

Single-cell technologies provide additional resolution by characterizing immune-cell populations individually. Single-cell transcriptomics and high-dimensional flow cytometry can identify changes within specific lymphocyte and myeloid populations and allow apoptotic, activation, and immune-checkpoint signals to be examined within defined cellular subsets. This is particularly relevant in sepsis, where immune responses may differ considerably between cell populations and between patients. Such approaches have improved the characterization of distinct immunological phenotypes that may be obscured in bulk analyses [45,46].

Integration of transcriptomic, proteomic, cellular, and clinical data into multi-omics models may further improve biological phenotyping and patient stratification. When combined with physiological monitoring and computational analysis, these datasets could eventually support a more dynamic assessment of immune dysfunction and help identify patient subgroups with different biological trajectories. At present, however, high cost, analytical complexity, limited standardization, and the need for specialized infrastructure restrict the use of these technologies largely to research settings [47].

No single biomarker or analytical method adequately captures the complexity of sepsis-associated cell death. Molecular and cellular markers provide the closest assessment of specific cell-death processes, whereas routine clinical indicators describe their broader immunological and organ-level context, and high-dimensional approaches characterize the heterogeneity of the host response. Their biological significance, potential clinical applications, principal limitations, and current implementation status are summarized in Table 3.

## 3. Limitations of Apoptosis Assessment

Apoptosis is recognized as a fundamental mechanism underlying immune dysregulation in sepsis, and numerous biomarkers have been proposed to characterize this process, including caspase activation, Annexin V staining, TUNEL assays, immune checkpoint molecules, and high-throughput transcriptomic and proteomic analyses. Although these techniques have considerably improved our understanding of sepsis pathophysiology, their translation into routine clinical practice remains limited.

Most methods require biological samples and specialized laboratory techniques, such as flow cytometry, molecular assays, or omics-based technologies, together with trained personnel and dedicated laboratory infrastructure. Furthermore, variations in sample collection, processing protocols, analytical platforms, and biomarker quantification reduce the reproducibility and comparability of results between institutions. An additional limitation is the relatively long turnaround time, which restricts their applicability in the intensive care setting, where therapeutic decisions frequently need to be made within hours. Consequently, despite the biological relevance of apoptosis in sepsis, its routine bedside assessment has not yet become feasible, emphasizing the need for rapid, standardized, and clinically applicable diagnostic technologies capable of providing real-time information on immune status [48,49].

## 4. Limitations of Conventional Clinical Evaluation

Despite substantial advances in sepsis management, accurate assessment of disease severity and immune status remains one of the greatest challenges in intensive care medicine. The implementation of the Sepsis Bundle has significantly improved patient management and contributed to reduced mortality; however, conventional clinical evaluation still provides only a partial representation of the complex biological processes underlying sepsis. Given the considerable interindividual variability in immune responses, inflammatory biomarkers and clinical severity scores are increasingly used to improve patient stratification and identify individuals who may benefit from personalized therapeutic strategies [50,51].

Current guidelines from the Surviving Sepsis Campaign recommend the use of standardized screening tools, with the SOFA score remaining the cornerstone for evaluating organ dysfunction according to the Sepsis-3 Consensus [2,52]. Additional prognostic models, including APACHE II, APACHE III, and APACHE IV, are widely used to estimate disease severity and predict clinical outcomes based on physiological variables collected at admission. Although these tools have demonstrated good predictive performance for mortality, they primarily quantify the extent of organ dysfunction rather than the underlying immune alterations driving disease progression. As a result, they represent static clinical snapshots and cannot fully capture the dynamic evolution of the host response throughout the septic episode [53].

Accurate diagnosis remains equally challenging. Although the Sepsis-3 definitions improved the characterization of sepsis and septic shock, no universally accepted diagnostic gold standard currently exists. Consequently, clinicians must rely on combinations of clinical findings, laboratory parameters, and severity scores to identify patients at risk. Simpler bedside tools, such as quick SOFA (qSOFA), facilitate early recognition but should not be considered definitive diagnostic instruments. Furthermore, microbiological confirmation is frequently unavailable at the time treatment is initiated, and blood cultures remain positive in only approximately 30–40% of patients with suspected sepsis [2,54].

Despite substantial advances in supportive care, numerous therapeutic strategies that demonstrated promising results in experimental models have failed to produce consistent benefits in clinical trials. One of the principal reasons is the marked biological heterogeneity of sepsis, in which patients with profoundly different immune phenotypes, including predominant hyperinflammation, apoptosis-driven immunosuppression, or mixed immune responses, are frequently evaluated and treated as a single clinical entity. Conventional clinical assessment, based primarily on organ dysfunction scores and routine laboratory investigations, is unable to adequately characterize these underlying biological differences or identify patients according to their immune status. Consequently, immunomodulatory therapies may be administered to biologically inappropriate patient populations, thereby reducing their apparent efficacy in clinical trials. These limitations highlight the need for integrated approaches capable of combining clinical variables, physiological signals, and molecular biomarkers of apoptosis and immune dysfunction to improve biological stratification, facilitate precision medicine, and identify patients most likely to benefit from targeted therapeutic interventions [55,56].

## 5. Physiological Signals and Their Relationship to Apoptosis

Although apoptosis can be evaluated using molecular and cellular biomarkers, its routine assessment in critically ill patients remains limited. In contrast, physiological signals are continuously available at the bedside and provide real-time information regarding the patient’s clinical status. Physiological signals encompass continuously acquired clinical, hemodynamic, respiratory, and laboratory variables whose temporal trajectories provide real-time insight into the evolving physiological state of the patient. While these variables do not directly measure apoptosis, they may represent downstream manifestations of the biological processes driven by immune dysregulation and organ injury.

### 5.1. Hemodynamic Signals

Hemodynamic signals provide early information regarding the progression of circulatory dysfunction during sepsis. Lactate kinetics reflect the complex metabolic and hemodynamic disturbances associated with sepsis, including impaired tissue perfusion, mitochondrial dysfunction, accelerated glycolysis, and altered lactate clearance. Increasing vasopressor requirements, persistent hypotension, and reduced heart rate variability indicate progressive cardiovascular dysfunction. Although these variables do not directly quantify apoptosis, they may represent downstream consequences of endothelial injury, microcirculatory impairment, mitochondrial dysfunction, and apoptosis-induced immune dysregulation that ultimately contribute to tissue hypoxia and multiple organ failure [57].

### 5.2. Respiratory Signals

Respiratory rate, worsening oxygenation indices, increasing ventilatory support requirements, prolonged mechanical ventilation, and changes in pulmonary mechanics reflect the progression of respiratory dysfunction and acute lung injury. These alterations arise from the combined effects of inflammation, endothelial damage, increased vascular permeability, and immune dysregulation, processes that are closely associated with apoptosis-mediated organ injury during sepsis [58].

### 5.3. Inflammatory and Hematological Signals

Inflammatory and hematological signals provide indirect information regarding the host immune response. Persistent fever or hypothermia reflects dysregulated inflammatory activity, whereas leukocyte trends, persistent lymphopenia, elevated neutrophil-to-lymphocyte ratio, and thrombocytopenia indicate evolving immune dysfunction and hematological impairment. Although these parameters are nonspecific, persistent lymphopenia and changes in leukocyte-derived inflammatory indices have been associated with apoptosis-induced immunosuppression and adverse clinical outcomes [42,59].

### 5.4. Organ Dysfunction Signals

Progressive organ dysfunction represents the cumulative clinical consequence of immune dysregulation and apoptosis during sepsis. Dynamic changes in SOFA score, together with the development of acute kidney injury, hepatic dysfunction, coagulation abnormalities, and worsening neurological status, reflect the evolution of multiple organ failure. Although these variables do not identify the underlying molecular mechanisms, they summarize the clinical impact of persistent inflammation, immune paralysis, endothelial injury, and apoptosis occurring throughout the course of sepsis [60,61].

Unlike apoptotic biomarkers, physiological signals cannot identify the molecular mechanisms responsible for disease progression. Instead, they reflect the clinical consequences of those mechanisms. Consequently, physiological monitoring and apoptotic biomarkers should be regarded as complementary rather than competing approaches. Physiological signals indicate that systemic deterioration is occurring, whereas apoptotic biomarkers provide insight into the biological processes responsible for immune dysfunction, immunoparalysis, and organ failure. Integrating these complementary sources of information has the potential to improve patient stratification, prognostic assessment, and individualized therapeutic decision-making [3,59].

Advances in continuous monitoring technologies now permit acquisition of large volumes of high-frequency physiological data. When integrated with molecular biomarkers of apoptosis and immune dysfunction, these multimodal datasets provide a more comprehensive representation of the biological and clinical evolution of sepsis than either approach alone. Such integrated approaches are particularly well suited to analyze these complex temporal relationships, facilitating earlier recognition of patient deterioration, improved risk stratification, and the development of precision medicine strategies in critically ill patients.

## 6. Biosensing Technologies and Multimodal Monitoring in Sepsis

### 6.1. Biosensors and Rapid Diagnostic Platforms

#### 6.1.1. Principles of Biosensing in Sepsis-Associated Cell Death

The assessment of apoptosis in sepsis still relies largely on laboratory techniques such as flow cytometry, immunoassays, and molecular assays, which require sample processing, specialized equipment, and trained personnel. Conventional laboratory assays often require centralized processing, specialized equipment, and prolonged turnaround times, limiting their utility in the early stages of sepsis when therapeutic decisions are highly time sensitive. Biosensors offer a different approach by coupling recognition of a biological target to an optical, electrical, or electrochemical signal that can be measured rapidly and, in some cases, at the point of care. Their potential value in sepsis lies not only in improved analytical sensitivity but also in shorter turnaround times, reduced sample volumes, and the possibility of serial or multiplex measurements, thereby facilitating timely clinical decision-making [62,63].

In the context of regulated cell death, however, the target being measured is important. Biosensors do not detect apoptosis or another cell-death pathway as a whole. Instead, they detect individual molecular events associated with these processes. Caspase activation and phosphatidylserine externalization are relatively direct indicators of apoptotic activity, whereas PD-1/PD-L1 expression provides information on immune exhaustion and its relationship with lymphocyte dysfunction. Similarly, caspase-1 and gasdermin D are more closely linked to pyroptosis, while HMGB1, TNF-α, IL-1β, and IL-18 reflect overlapping processes involving inflammation, cellular injury, and regulated cell death. These differences need to be considered when interpreting a biosensor signal, particularly in sepsis, where several pathways may be active simultaneously, in order to distinguish the underlying mechanisms of regulated cell death [64,65,66].

A further distinction concerns the maturity of the available technologies. Biosensors capable of detecting apoptosis-specific targets have been described, but many have been developed in experimental systems or for applications outside sepsis. Conversely, several point-of-care platforms have already been tested using samples from patients with sepsis, but most measure inflammatory or host-response biomarkers rather than apoptosis itself. Thus, analytical feasibility should not be equated with clinical validation in sepsis. Representative biosensing platforms, together with their main analytical characteristics and stage of validation, are summarized in Table 4. 

#### 6.1.2. Biosensing Technologies and Representative Platforms

Clinically useful biosensing platforms should combine analytical accuracy with rapid processing, accessibility, cost-effectiveness, and compatibility with point-of-care testing. A broad range of technologies has been investigated for sepsis applications, including electrochemical and optical biosensors, immunosensors, nanoparticle-based systems, microfluidic devices, and multiplex platforms. Their targets range from inflammatory and host-response biomarkers, such as IL-6, TNF-α, CRP, and procalcitonin, to molecules more directly associated with regulated cell death, including activated caspases. The following examples illustrate the different sensing strategies, analytical performance, and stages of development currently reported in this field [63,73].

Electrochemical biosensors are particularly attractive for point-of-care applications because they can be miniaturized, require small sample volumes, and convert biomolecular recognition events directly into measurable electrical signals. Depending on the design, target binding can be detected through changes in current, potential, impedance, or voltammetric response. These characteristics have encouraged the development of electrochemical platforms for both apoptosis-associated biomarkers and inflammatory mediators relevant to sepsis. However, their performance in complex biological samples may be affected by electrode fouling, matrix interference, surface stability, and differences between sensor batches [66,73].

A direct application to apoptosis monitoring was reported by Shin et al., who developed an electrochemical biosensor for caspase-3 using an affinity peptide-displaying M13 bacteriophage as the recognition element. The phage particles were immobilized on a gold surface, and caspase-3 binding was evaluated by cyclic and square-wave voltammetry. The sensor reached a limit of detection of 0.39 μM and was able to detect changes in caspase-3 activity in apoptotic HeLa cells. The use of an affinity phage offers an alternative to conventional antibody-based recognition and demonstrates that an executioner caspase can be detected electrochemically. Nevertheless, this remains a proof-of-concept approach: it was evaluated in a cellular model and has not been tested in blood samples or in patients with sepsis [67].

Optical biosensors detect biomolecular interactions through changes in optical signals, using approaches such as fluorescence, surface plasmon resonance (SPR), and surface-enhanced Raman spectroscopy. Their high sensitivity and capacity for label-based or label-free detection make them attractive for rapid biomarker assessment in sepsis. Tao et al. developed a fluorescence biosensor for caspase-8 based on a fluorophore-labeled peptide substrate and reduced graphene oxide (rGO), which acts as a fluorescence quencher. Cleavage of the peptide by caspase-8 releases the fluorophore-containing fragment from the rGO surface, restoring the fluorescence signal. The assay showed a linear response between 0.2 and 5 U/mL and a limit of detection of 0.2 U/mL and was subsequently used to monitor intracellular caspase-8 activity. Although the platform combines enzyme-specific recognition with the signal-enhancing properties of a nanomaterial, its application has so far been limited to experimental and cellular models rather than clinical samples from patients with sepsis [68].

Optical platforms have also been developed for biomarkers associated with cellular injury and inflammation. Chiang et al. described a fiber-optic localized surface plasmon resonance biosensor for HMGB1 based on a carboxymethyl-dextran-modified gold nanoparticle surface. The sensor achieved a limit of detection of 43.4 pg/mL, with a linear range of 10^−10^–10^−6^ g/mL and a response time below 10 min. The fiber-optic configuration and gold nanoparticle surface allow rapid, label-free detection, but the biological meaning of the measured target must be considered. HMGB1 is a damage-associated molecular pattern released in several forms of cellular stress and injury and is not specific for apoptosis. Moreover, the reported analytical performance has not yet been translated into validation of the platform for monitoring regulated cell death in septic patients [69].

Optical biosensors have also been extensively investigated for sepsis-related inflammatory biomarkers, including IL-6, IL-1β, TNF-α, CRP, and procalcitonin. However, many cell-death-specific applications remain experimental, and instrumentation requirements, interference from complex biological samples, and limited clinical validation currently restrict their routine use [68,73,74].

Immunosensors use antigen–antibody recognition and may employ electrochemical, optical, or other transduction strategies; therefore, they overlap with several technological categories rather than representing a mutually exclusive sensing modality. Sánchez-Tirado et al. developed a dual electrochemical immunosensor for IL-1β and TNF-α using screen-printed carbon electrodes modified with functionalized double-walled carbon nanotubes. The assay showed linear ranges of 0.5–100 pg/mL for IL-1β and 1–200 pg/mL for TNF-α, with limits of detection of 0.38 and 0.85 pg/mL, respectively. It was applied to spiked human serum and real saliva samples. The use of disposable electrodes and simultaneous detection of two cytokines are useful features for point-of-care applications, although neither IL-1β nor TNF-α is specific for apoptosis, and the device itself has not been validated in a sepsis cohort [70].

PD-L1 is another relevant target because of its relationship with immune dysfunction in sepsis, although available biosensing technologies for PD-L1 have largely been developed in oncology. Du et al. reported an electrochemical biosensor based on electrodes modified with molybdenum disulfide and multi-walled carbon nanotubes, together with a PD-L1 antibody–quantum dot conjugate. The combination of nanomaterials and antibody-based recognition illustrates how immune-checkpoint proteins can be incorporated into biosensing systems. From the perspective of sepsis, however, this technology should be considered a proof of analytical feasibility rather than a clinically validated approach: the platform was designed to assess tumor immunotherapy and has not been evaluated for circulating PD-L1 measurements in septic patients [75].

This high molecular specificity of immunosensors enables sensitive detection of circulating proteins associated with inflammation, immune dysfunction, and regulated cell death, and their compatibility with miniaturized and multiplex formats makes them particularly attractive for point-of-care applications. However, antibody stability, cross-reactivity, matrix interference, assay reproducibility, and the limited clinical validation of cell-death-specific applications remain important barriers to routine implementation [63,74].

Nanoparticle-based biosensors exploit the optical, electrical, magnetic, or catalytic properties of nanomaterials to enhance biomarker capture and signal amplification, thereby enabling highly sensitive detection from small biological samples. Gold nanoparticles, magnetic nanoparticles, quantum dots, and other nanostructured materials have been incorporated into sensing platforms for inflammatory and cell-death-related biomarkers, often with the aim of increasing analytical sensitivity while reducing sample volume and assay time [76]. Alba-Patiño et al., for example, developed a paper-based plasmonic biosensor for IL-6 using antibody-functionalized gold nanoparticles as colorimetric nanoprobes. The signal was quantified with a smartphone application, allowing IL-6 detection in unprocessed whole blood with a limit of detection of 0.1 pg/mL and a total assay time below 17 min [71]. Other nanomaterial-based approaches include CuInS_2_/ZnS nanocrystal fluoroimmunoassays for IL-6 and gold nanoparticle-based nanoplasmonic platforms for multiplex cytokine detection [76]. These examples demonstrate how nanomaterials can enhance signal generation and facilitate portable or multiplex analysis; however, the biomarkers measured are predominantly inflammatory rather than direct indicators of regulated cell death. Most nanoparticle-based platforms also remain investigational, with reproducibility, nanomaterial stability, manufacturing standardization, and clinical validation representing important barriers to translation [69,71,76].

Microfluidic and lab-on-chip platforms integrate sample handling, processing, and biomarker detection within miniaturized devices, reducing sample and reagent requirements while allowing several analytical steps to be performed on a single platform. In sepsis, these platforms have been investigated for multiplex detection of inflammatory mediators such as IL-6, IL-8, IL-10, and TNF-α and can be combined with flow cytometry or other cellular-analysis approaches to characterize immune-cell populations and cell-death-associated biomarkers [77,78]. Valera et al. developed a microfluidic biochip that combines antigen-specific capture chambers, microscale bead-based immunodetection, and electrical Coulter counting for protein quantification in undiluted human plasma. The platform was evaluated for IL-6 measurement in 29 plasma samples and showed good correlation with the reference method (R^2^ = 0.81). An important feature of this approach is its potential to combine protein and cellular measurements within the same microfluidic system, which could be useful for simultaneous assessment of soluble inflammatory mediators and immune-cell alterations in sepsis [79]. More recently, a multiplex microfluidic aptasensor based on porous silicon optical transducers and reflectometric interference Fourier transform spectroscopy was developed for simultaneous detection of IL-6, TNF-α, and CRP. The device incorporates three biosensing zones within a single microfluidic chip and uses immobilized aptamers for label-free detection, with signal variation below 7% between individual and multiplex measurements [72]. These platforms illustrate the potential of microfluidics to move from single-analyte measurement toward integrated sepsis profiling; however, the biomarkers evaluated remain primarily inflammatory rather than specific indicators of regulated cell death, and further validation in clinical sepsis cohorts is required before routine implementation.

Multiplex point-of-care platforms enable the simultaneous measurement of multiple biomarkers from a single, low-volume biological sample, providing a more comprehensive representation of the heterogeneous host response to sepsis than single-analyte assays. Tanak et al. developed the DETecT Sepsis electrochemical point-of-care platform for the simultaneous measurement of IL-6, IL-8, IL-10, TRAIL, and IP-10 using EnLiSense’s READ platform and a disposable sensor cartridge containing nanofilm semiconductor/metal electrode interfaces functionalized with specific capture probes. Detection was based on non-faradaic electrochemical impedance spectroscopy. The platform required less than 40 μL of undiluted plasma, provided results within approximately 5 min, and achieved detection limits in the pg/mL range. In an initial evaluation of 40 clinical samples, measurements showed strong agreement with reference methods (Pearson’s r > 0.90). Although this platform represents a more advanced stage of clinical translation than most apoptosis-specific biosensors, the measured biomarkers primarily characterize the inflammatory and immune response to sepsis rather than apoptosis itself [66]. Such platforms are particularly relevant to sepsis-associated cell death, where integration of inflammatory, immune, and cell-death-related biomarkers may ultimately provide a more informative molecular profile than isolated measurements. However, analytical complexity, cross-reactivity, cost, assay standardization, and the need for broader prospective clinical validation remain important barriers to routine implementation [80,81].

Despite advances in biosensor design, a gap remains between analytical performance and clinical validation. Direct sensing of apoptosis-associated targets such as caspases is technically feasible but remains largely experimental, whereas sepsis-specific platforms are closer to point-of-care use but predominantly measure inflammatory or host-response biomarkers. Future studies should validate cell-death-associated targets in clinical samples and determine their added value beyond established biomarkers and severity scores.

### 6.2. Multiplex Biosensing and Multimodal Monitoring

The heterogeneous nature of sepsis makes it unlikely that a single biomarker can adequately reflect the biological changes occurring during the course of the disease. Multiplex biosensing offers the possibility of measuring several complementary targets within the same analytical platform, allowing apoptosis-associated biomarkers to be assessed together with indicators of inflammation, immune dysfunction, and tissue injury. Activated caspases or phosphatidylserine exposure, for example, could be interpreted alongside inflammatory mediators such as IL-6, IL-1β, and TNF-α or immune-related markers such as PD-1/PD-L1. Such combinations may provide a broader picture of the patient’s biological status than individual measurements, particularly when repeated over time.

Nanomaterials can improve the performance of these platforms by increasing the available sensing surface, enhancing biomolecule immobilization, and amplifying optical or electrochemical signals. The examples described above illustrate different ways in which these properties can be exploited: reduced graphene oxide has been used for fluorescence-based detection of caspase-8, gold nanoparticles for optical or colorimetric detection of HMGB1 and IL-6, and carbon nanotubes for simultaneous electrochemical measurement of IL-1β and TNF-α. Quantum dots and nanoplasmonic structures offer further possibilities for signal enhancement and parallel detection of several targets. These characteristics make nanomaterials particularly useful for miniaturized and multiplex biosensors, although improved analytical sensitivity must be balanced against challenges related to reproducibility, surface functionalization, matrix interference, manufacturing standardization, and clinical validation [68,69,70,74,75,76].

Multiplex biosensing can also form part of a wider multimodal monitoring strategy in which molecular measurements are considered together with routinely acquired physiological and clinical information. Continuous or serial variables such as lactate kinetics, vasopressor requirements, heart rate variability, respiratory parameters, temperature patterns, and hemodynamic changes can be integrated with biomarkers of inflammation, immune dysfunction, apoptosis, and endothelial or organ injury. These different types of data provide complementary information: molecular and cellular biomarkers may offer insight into the biological processes occurring during sepsis, whereas physiological variables and organ dysfunction scores reflect their clinical consequences. Their combined assessment may therefore provide a more complete representation of disease progression than either approach alone [80].

The integration of multiplex biomarker measurements with high-frequency bedside data also creates analytical challenges. Serial monitoring can generate large and heterogeneous datasets in which clinically relevant relationships may be difficult to recognize using conventional analysis alone. Computational approaches may help identify temporal patterns between changes in biomarker profiles, physiological deterioration, and clinical outcomes, potentially supporting more dynamic patient stratification. This provides a direct link between biosensing and the artificial intelligence approaches discussed in the following section, where machine-learning methods may facilitate interpretation of complex biosensor outputs and their integration with physiological and clinical data [81,82].

The integration of biomarker recognition, signal transduction, nanomaterial-assisted enhancement, and multiplex detection provides a framework for linking molecular biosensing with physiological and clinical monitoring. The principal components of this biomarker–biosensor interface and their progression toward multimodal data integration and AI-assisted analysis are summarized in Figure 2.

## 7. Artificial Intelligence for Biosensing and Multimodal Sepsis Monitoring

### 7.1. AI-Assisted Biosensing and Signal Interpretation

The increasing use of multiplex biosensors in sepsis creates analytical challenges that extend beyond biomarker detection itself. A single platform may generate simultaneous measurements of several molecular targets, while repeated sampling produces temporal profiles that must be interpreted together with physiological and clinical changes. Machine-learning methods are particularly suited to this type of analysis because they can identify patterns and relationships across multiple variables without requiring each signal to be interpreted independently. In biosensing applications, these methods can assist with signal processing, classification of complex sensor responses, correction of background interference, and interpretation of multiplex biomarker profiles, potentially reducing the time between sample acquisition and clinically useful information [83,84,85].

This approach is particularly relevant to sepsis-associated cell death, where the biological meaning of a biosensor signal depends strongly on the combination of markers being measured. Caspase activation or phosphatidylserine exposure may provide relatively direct evidence of apoptotic activity, whereas inflammatory cytokines, PD-1/PD-L1, lymphocyte counts, and organ-injury biomarkers provide complementary information on the accompanying immune and systemic response. Rather than assigning diagnostic significance to a single measurement, artificial intelligence (AI)-assisted platforms could analyze these signals jointly and identify biomarker patterns associated with different biological states or disease trajectories. Recent reviews of sepsis biosensing have therefore highlighted the integration of biosensors with computational and AI-based analysis as an important direction for the development of rapid diagnostic and monitoring platforms [83].

### 7.2. Integration of Biosensor Outputs with Physiological and Clinical Data

The potential value of AI becomes greater when biosensor-derived measurements are combined with the continuous information already available in the intensive care unit. Heart rate and its variability, blood pressure, respiratory parameters, temperature, vasopressor requirements, laboratory results, and organ dysfunction scores provide information on the clinical consequences of sepsis, whereas molecular and cellular biomarkers can provide insight into the underlying biological processes. Machine-learning models can integrate these heterogeneous data streams and examine their evolution over time, allowing changes in biomarker profiles to be interpreted in the context of simultaneous physiological deterioration or recovery [86,87,88,89].

Existing AI models for sepsis illustrate the feasibility of analyzing complex longitudinal clinical data, although most were not developed specifically for biosensor outputs. The Sepsis Early Risk Assessment approach uses routinely available clinical information for early sepsis prediction, while Deep Artificial Intelligence Sepsis Expert applies a recurrent neural survival model to time-series clinical data and can generate continuously updated estimates of sepsis risk [85,87]. These systems demonstrate how temporal relationships that may be difficult to recognize from individual measurements can be extracted computationally. Their relevance to biosensing lies in the possibility of extending the same principle to serial molecular measurements, allowing rapidly acquired biomarker data to become additional inputs rather than isolated laboratory results [89,90].

More recent multimodal approaches further support this concept. Computational models have been developed to represent heterogeneous sepsis data within a common analytical framework, while multimodal learning approaches have combined different sources of clinical information for sepsis detection and risk assessment [81,82,88]. Although these systems do not yet provide direct evidence that apoptosis-specific biosensor measurements improve sepsis prediction, they provide a framework into which such measurements could eventually be incorporated. Future models could combine serial caspase activity, phosphatidylserine exposure, PD-1/PD-L1, inflammatory mediators, or other biosensor-derived signals with physiological trajectories and conventional laboratory variables. This distinction is important because the technological capacity for multimodal integration is developing more rapidly than the clinical validation of apoptosis-specific biosensors in patients with sepsis.

### 7.3. From Multiplex Biosensing to Patient Stratification 

An important potential application of this combined approach is the identification of biologically distinct patient profiles. Sepsis encompasses markedly different immune states, ranging from predominant inflammation to profound immunosuppression, and these states may coexist or change during the course of illness. Analysis of multiplex biomarker patterns together with physiological trajectories could therefore provide more information than conventional severity scores or isolated biomarkers alone. Machine-learning methods may help recognize combinations of cell-death-associated, inflammatory, immune, and organ-dysfunction signals that characterize particular disease trajectories and support more refined patient stratification [89,90,91].

However, this application remains largely prospective. Current AI systems for sepsis predominantly rely on electronic health records, conventional laboratory variables, and physiological data, while direct integration of apoptosis-associated biosensor measurements has not yet undergone sufficient clinical validation. The immediate role of AI should therefore be viewed as a means of improving the interpretation and integration of increasingly complex biosensor and multimodal datasets rather than as an established method for identifying apoptosis-driven sepsis phenotypes. Prospective studies will be needed to determine whether adding cell-death-associated biosensor signals improves diagnostic or prognostic performance beyond existing clinical, physiological, and inflammatory markers.

## 8. Ethical Considerations

The integration of apoptosis-associated biomarkers, biosensing technologies, physiological monitoring, and artificial intelligence into sepsis assessment offers new opportunities for earlier diagnosis, improved risk stratification, and personalized therapeutic decision-making. However, many of these approaches remain investigational, and their clinical use requires careful evaluation of analytical reliability, clinical validity, and the consequences of basing decisions on incompletely validated measurements or predictive models. These considerations become particularly important when molecular and physiological data are combined to support patient stratification or clinical decision-making [89].

Artificial intelligence algorithms are highly dependent on the quality, completeness, and representativeness of the data used for their development. Missing data, inconsistent clinical documentation, and institutional differences in patient populations may introduce bias and reduce model performance when applied to external cohorts [85]. Consequently, many prediction models that perform well during internal validation demonstrate lower accuracy when evaluated in independent populations, highlighting the need for robust multicenter validation before clinical implementation [89,90]. Similar caution is required when interpreting apoptosis-associated biomarkers. Caspases, phosphatidylserine exposure, PD-1/PD-L1, and lymphopenia reflect different aspects of cell death and immune dysfunction, and none should be used in isolation to guide clinical decisions. Their interpretation should remain linked to physiological monitoring, conventional laboratory findings, and the overall clinical context. Reliance on insufficiently validated biomarkers or prediction models may result in inappropriate patient stratification and potentially unsuitable therapeutic interventions [92].

Data privacy, algorithmic transparency, and clinical responsibility represent additional concerns. Multimodal monitoring systems may combine molecular measurements with continuously acquired physiological and clinical data, increasing both the volume and complexity of patient information being processed. Limited interpretability of AI-based models may make it difficult for clinicians to understand how individual variables contribute to a prediction or recommendation. For this reason, AI-assisted analytical platforms should be regarded as decision-support tools rather than substitutes for clinical judgment. Responsibility for diagnosis and treatment must remain with the treating clinician, who should integrate algorithmic outputs with biological, physiological, and clinical information when making therapeutic decisions [93,94].

Accessibility is also relevant to the clinical translation of these technologies. Advanced biosensing and multimodal monitoring systems may require specialized analytical platforms, computational infrastructure, trained personnel, and additional financial resources. These requirements may limit their implementation outside well-resourced academic or tertiary centers and are particularly relevant in low- and middle-income settings, where the burden of sepsis remains high [1]. Future development should therefore consider not only analytical performance but also cost, scalability, standardization, ease of use, and compatibility with existing clinical workflows. Addressing these practical barriers will be important if advances in biosensing and multimodal monitoring are to be evaluated and implemented across different healthcare settings [95,96].

## 9. Knowledge Gaps and Priorities for Clinical Translation

Despite substantial progress in sepsis biomarker research, the transition from biological relevance to clinical utility remains difficult. This is particularly evident for apoptosis-associated biomarkers. Activated caspases and phosphatidylserine externalization are attractive because they reflect events relatively close to the cell-death process, yet their value as longitudinal biomarkers in patients with sepsis remains insufficiently established. At the same time, many biosensing platforms report excellent analytical sensitivity but have been evaluated mainly in buffer, spiked samples, or experimental models rather than in samples obtained from well-characterized sepsis cohorts. Analytical performance should therefore be considered separately from biological relevance and clinical usefulness, and future studies should address all three levels of validation [64,97].

The temporal dimension represents another important knowledge gap. Sepsis is heterogeneous and evolves through changing inflammatory and immunosuppressive states, while the molecular events associated with apoptosis do not necessarily occur simultaneously. Consequently, the clinical meaning of an apoptosis-associated biomarker is likely to depend on when it is measured and on the immune state of the patient at that time. Single measurements may overlook these changes and partly explain inconsistent biomarker–outcome associations across heterogeneous sepsis populations. Future studies should therefore use predefined serial sampling and examine biomarker trajectories rather than relying exclusively on isolated values. Ideally, changes in apoptosis-associated markers should be analyzed alongside immune-cell counts, inflammatory biomarkers, physiological variables, and evolving organ dysfunction to determine whether they precede, accompany, or follow clinical deterioration [97].

A clinically realistic strategy is unlikely to depend on a single apoptosis-associated biomarker. A more informative panel could combine a relatively direct marker of apoptosis, such as caspase-3 activity or phosphatidylserine exposure, with an indicator of immune dysfunction, such as PD-1/PD-L1 or persistent lymphopenia, and an established inflammatory marker such as IL-6 or procalcitonin. These measurements could then be interpreted together with routinely available variables, including lactate kinetics and dynamic SOFA. The purpose of such a panel would not simply be to increase the number of measured biomarkers but to capture complementary aspects of the disease process: apoptotic activity, immune status, systemic inflammation, and the resulting physiological and organ dysfunction. The growing development of multiplex biosensors makes this type of combined assessment technically plausible, although its clinical benefit remains to be demonstrated [64,72,82].

This framework also generates hypotheses that can be tested prospectively. Serial changes in caspase activity or phosphatidylserine exposure may precede persistent lymphocyte depletion or identify patients progressing toward a predominantly immunosuppressive phenotype. Apoptosis-associated measurements may also distinguish patients with comparable conventional severity scores but different biological trajectories. Most importantly, adding these measurements to inflammatory biomarkers and physiological data should be tested against the alternative hypothesis that they provide little or no additional clinically useful information. Demonstrating an association with outcome alone would therefore be insufficient; the relevant question is whether apoptosis-associated measurements improve stratification or prediction beyond existing clinical and laboratory information [82,97].

Clinical translation will require a staged validation strategy. Candidate biosensors should first demonstrate reproducibility, selectivity, stability, resistance to interference, and reliable performance in clinically relevant matrices such as whole blood or plasma. Subsequent prospective studies should enroll clearly characterized patients with sepsis, use standardized sampling intervals, and include simultaneous measurements using established reference methods. Multicenter studies will ultimately be required to account for differences in patient populations, pathogens, clinical management, and laboratory practice. Importantly, extremely low limits of detection should not be regarded as evidence of clinical superiority in themselves; sensitivity must be balanced against assay complexity, robustness, turnaround time, sample preparation, and compatibility with bedside use [64,82].

The same principle applies when apoptosis-associated biosensor measurements are incorporated into multimodal or machine-learning models. New models should be compared directly with otherwise equivalent models based only on conventional physiological, clinical, and laboratory variables, allowing the incremental contribution of cell-death-associated information to be quantified. Evaluation should extend beyond discrimination metrics such as the area under the receiver operating characteristic curve and include calibration, clinical utility, robustness to missing data, and external validation. Recent studies of sepsis prediction models have shown that performance observed during model development does not necessarily translate into equivalent clinical utility during external validation, reinforcing the need for independent and multicenter evaluation before implementation [98,99].

Ultimately, the key translational question is not whether apoptosis can be detected with increasing analytical sensitivity, but whether measuring it improves the assessment of patients with sepsis. A clinically useful platform should provide information that cannot be obtained as readily from established inflammatory biomarkers, physiological monitoring, or organ dysfunction scores, while remaining rapid and practical enough for repeated measurements in critically ill patients. Prospective studies that combine serial apoptosis-associated measurements with multiplex biosensing, longitudinal physiological monitoring, and appropriately validated analytical models will therefore be necessary to determine whether the proposed framework offers genuine incremental value in patient stratification and clinical decision-making [82,97].

## 10. Conclusions

The main value of biosensing in sepsis may lie not only in the sensitive detection of apoptosis-associated biomarkers but also in the possibility of measuring them repeatedly as the patient’s condition evolves. At present, the dynamics of cell death are difficult to follow in clinical practice, while conventional biomarkers and severity scores mainly reflect inflammation and the consequences of organ dysfunction. Rapid biosensing platforms requiring small sample volumes could make serial assessment more practical and could allow apoptosis-associated signals to be measured together with complementary markers of immune dysfunction and inflammation.

An important question is whether these measurements would provide useful information beyond what is already available at the bedside. Relatively direct markers of apoptosis, such as caspase activity or phosphatidylserine externalization, are unlikely to be informative when considered in isolation. Their value may be greater when changes over time are interpreted alongside immune-related biomarkers, physiological trends, and the progression or recovery of organ dysfunction. Combining these different sources of information within a multimodal monitoring approach, with machine-learning methods used where appropriate, could help relate changes in cell-death activity to the patient’s changing clinical and immune status.

Whether apoptosis-associated biosensing actually adds clinically useful information, however, remains to be established. Future studies should therefore look beyond analytical performance or associations between individual biomarkers and outcome. The more relevant question is whether serial measurement of cell-death-associated biomarkers improves patient stratification, prognostic assessment, or the identification of evolving immune dysfunction when compared with currently available clinical and laboratory tools. Addressing this question will require prospective studies with serial sampling, carefully selected biomarker combinations, direct comparison with conventional monitoring, and ultimately validation in independent multicenter cohorts.

## Figures and Tables

**Figure 1:**
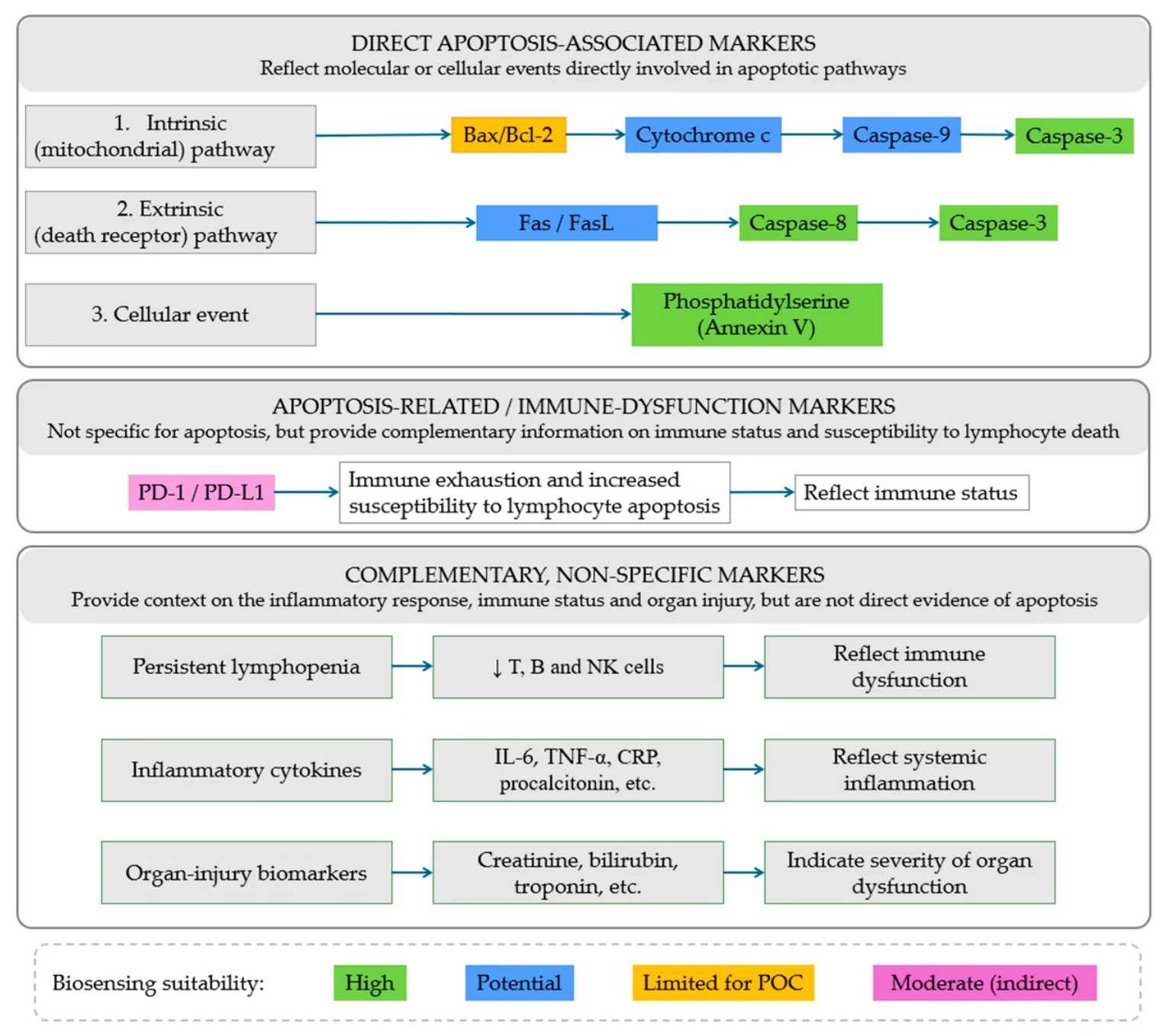
Apoptosis-associated biomarkers in sepsis: biological relevance and suitability for biosensing. Legend: Biosensing suitability—high (green) for caspase-3, caspase-8, and phosphatidylserine; potential (blue) for cytochrome c, caspase-9, and Fas/Fas ligand (FasL); limited for point-of-care (POC) applications (yellow) for Bax/Bcl-2; and technically suitable but indirect (purple) for PD-1/PD-L1.

**Figure 2:**
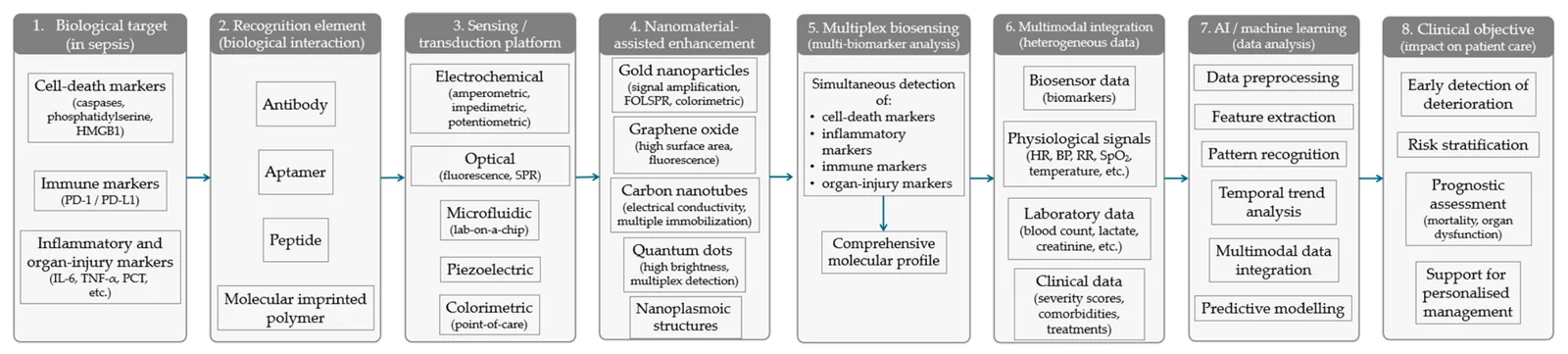
Biosensing strategies for sepsis-associated cell-death biomarkers and their integration into multiplex and multimodal monitoring.

**Table 1:** Clinical indicators of organ dysfunction providing complementary context to regulated cell death in sepsis.

Organ/System	Biomarker(s)	Clinical Significance	Relationship with Regulated Cell Death
Liver	alanine aminotransferase (ALT), aspartate aminotransferase (AST), bilirubin	Hepatocellular injury and cholestatic dysfunction	Indirect indicators of liver injury resulting from inflammation, microcirculatory dysfunction, mitochondrial injury, apoptosis, and other regulated cell death pathways
Myocardium	Cardiac troponin T (or I), N-terminal pro-B-type natriuretic peptide (NT-proBNP)	Septic cardiomyopathy and myocardial dysfunction	Reflect cardiomyocyte injury secondary to inflammation, apoptosis, oxidative stress, and microvascular dysfunction
Kidney	Creatinine	Acute kidney injury	Indicates renal dysfunction associated with inflammation, tubular injury, apoptosis, and impaired perfusion
Systemic dysfunction	Lactate	Global metabolic derangement	Reflects impaired tissue perfusion, mitochondrial dysfunction, accelerated glycolysis, and altered lactate clearance
Hematologic system	Platelet count	Coagulopathy and endothelial activation	Thrombocytopenia reflects platelet consumption, endothelial injury, immune dysregulation, and systemic inflammation
Global severity	Dynamic Sequential Organ Failure Assessment (SOFA) score	Overall organ dysfunction and prognosis	Integrates dysfunction across multiple organs and indirectly reflects the cumulative consequences of regulated cell death and systemic inflammation

**Table 2:** Clinical studies linking cell-death-associated and organ-injury biomarkers with outcomes in sepsis.

Study	Population	Biomarker	Clinical Endpoint	Main Finding
Drewry et al., 2014 [12]	335 adults with bacteremia and sepsis	Persistent lymphopenia	28-day mortality, 1-year mortality, secondary infection	Day-4 absolute lymphocyte count was independently associated with 28-day survival (adjusted OR 0.68, 95% CI 0.51–0.91) and 1-year survival (adjusted OR 0.74, 95% CI 0.59–0.93). Severe persistent lymphopenia was also associated with secondary infections.
Jing et al., 2024 [38]	1306 adult septic patients with persistent and nonpersistent lymphopenia	Persistent lymphopenia	28-day and 90-day mortality, secondary infection, disease severity	913 (69.9%) patients developed persistent lymphopenia. Compared to nonpersistent lymphopenia, persistent lymphopenia was associated with higher mortality (28 d mortality (38.0% vs. 22.9%, *p* < 0.05), and 90 d mortality (51.2% vs. 31.3%, *p* < 0.05)), poorer clinical condition, increased risk of secondary infection, and increasing SOFA scores as lymphopenia persisted (8.0 (6.0–10.0) vs. 6.0 (4.0–8.0), *p* < 0.05).
Tang et al., 2024 [39]	100 adults with sepsis; 89 healthy controls	PD-1^+^ natural killer (NK) cells	28-day mortality	PD-1^+^ NK cells were higher in non-survivors than survivors (15.1% vs. 8.6%, *p* < 0.01). Combined with SOFA, PD-1^+^ NK cells predicted 28-day mortality (AUC = 0.91, 95%CI 0.82–0.99); high PD-1^+^ NK-cell proportion was associated with mortality (aHR 1.34, 95% CI 1.19–1.50).
Wang et al., 2020 [40]	128 trauma-induced patients with sepsis, peripheral blood mononuclear cell (PBMC) analysis	Caspase-1-dependent PBMC pyroptosis	Disease severity, mortality	The proportion of pyroptotic PBMCs correlated positively with Acute Physiology And Chronic Health Evaluation (APACHE) II and SOFA scores (*p* < 0.01) and was associated with increased 28-day mortality (aHR 1.234, 95% CI 1.014–1.502), supporting a relationship between caspase-1-dependent pyroptosis and sepsis severity.
Jendoubi et al., 2019 [41]	75 adults with septic shock	High-sensitivity cardiac troponin I (hs-cTnI)	28-day mortality	hs-cTnI was elevated in 47% of patients; 28-day mortality was 54.6%. hs-cTnI concentrations were higher in non-survivors at serial time points, and the 72 h value was independently associated with 28-day mortality.
Choi & Lee, 2024 [42]	418 adults with septic shock	NT-proBNP, lactate, blood urea nitrogen (BUN), creatinine, C-reactive protein (CRP), procalcitonin	In-hospital mortality	Lactate, NT-proBNP and BUN differed significantly between survivors and non-survivors. The AUC values for predicting in-hospital mortality were as follows: Lactate, 0.678 (95% CI: 0.627–0.726); NT-proBNP, 0.648 (95% CI: 0.600–0.694); BUN, 0.630 (95% CI: 0.582–0.676).

**Table 3:** Biological and analytical characteristics of candidate apoptosis-associated biomarkers in sepsis.

Biomarker	Relationship to Apoptosis	Biological Specificity	Temporal Characteristics	Accessibility/Sample	Prognostic Evidence in Sepsis	Suitability for Biosensing
Caspase-3	Executioner caspase; reflects downstream activation of apoptosis	Relatively high, but activation may occur in different cell types and is not sepsis-specific	Associated with the execution phase; may provide a relatively narrow detection window	Cellular/tissue assessment; circulating forms/activity potentially measurable	Experimental and limited clinical evidence; not established for routine prognostication	High analytical interest; direct electrochemical sensing has been demonstrated, but not clinically validated in sepsis
Caspase-8	Initiator caspase of the extrinsic pathway	Relatively high for death-receptor signaling, although also involved in crosstalk with other RCD pathways	Earlier/upstream event than executioner caspase-3	Primarily cellular/tissue; activity can be measured experimentally	Clinical prognostic evidence in sepsis remains limited	Promising; fluorescence peptide-based sensing demonstrated experimentally
Caspase-9	Initiator caspase of the mitochondrial pathway	Relatively high for intrinsic apoptotic signaling	Early/intermediate event preceding executioner caspase activation	Mainly cellular/tissue assays	Limited clinical prognostic validation in sepsis	Technically detectable, but limited sepsis-specific biosensor development
Phosphatidylserine externalization	Early membrane event associated with apoptosis	Moderate; PS exposure is not exclusively apoptotic	Generally occurs early, before loss of membrane integrity	Primarily cell-associated; detectable by Annexin V-based methods; also present on extracellular vesicles	Biological relevance established, but limited evidence as an independent circulating prognostic marker	Good, particularly for optical/affinity-based detection, although specificity requires complementary markers
Fas/FasL	Activation of extrinsic death-receptor signaling	Moderate; reflects death-receptor activation but is not exclusively indicative of completed apoptosis	Upstream/early signaling event	Membrane expression and soluble circulating forms	Associations with sepsis severity/outcome reported, but clinical utility remains uncertain	Potentially suitable for antibody/aptamer-based sensing
Cytochrome c	Released following mitochondrial outer-membrane permeabilization	Relatively high for mitochondrial injury/apoptotic signaling, but mitochondrial damage can occur outside apoptosis	Intermediate event before caspase-9/-3 activation	Circulating cytochrome c can potentially be measured in plasma/serum	Some prognostic evidence, but insufficient for routine use	Potentially amenable to immunosensing; limited sepsis-specific biosensor validation
Bcl-2/Bax ratio	Reflects balance between anti- and pro-apoptotic mitochondrial signaling	Mechanistically informative but not a circulating biomarker in the conventional sense	Reflects regulation preceding mitochondrial permeabilization	Requires cellular/tissue analysis	Research associations; limited practical prognostic application	Low for routine point-of-care biosensing because intracellular quantification is required
PD-1/PD-L1	Associated with immune exhaustion and increased susceptibility to apoptosis; not a direct apoptosis marker	Low for apoptosis specifically	May persist during sepsis-associated immunosuppression	Cell-surface expression; soluble forms can be measured in blood	Clinical associations with immune dysfunction, secondary infection and adverse outcomes	Good technical suitability for affinity-based biosensors, but biological interpretation is less apoptosis-specific

**Table 4:** Comparative characteristics of representative biosensing approaches for biomarkers relevant to sepsis-associated cell death. All abbreviations used in the table can be found in the Abbreviations section.

Biosensing Approach	Representative Target(s)/Study	Recognition Element	Transduction Mechanism/Sensing Material	Sample Matrix	Limit of Detection	Linear Range	Analysis Time	Multiplexing Capability	Validation Stage
Electrochemical biosensor	Caspase-3; [67]	Caspase-3 affinity peptide displayed on M13 bacteriophage	Electrochemical; phage-modified gold electrode, cyclic and square-wave voltammetry	Purified caspase-3; apoptotic HeLa-cell model	0.39 μM	NR	<15 h	No	Proof-of-concept/cellular testing; not evaluated in clinical sepsis samples
Optical biosensor–fluorescence	Caspase-8; [68]	Fluorophore-labeled peptide substrate	Fluorescence; reduced graphene oxide (rGO) quencher	In vitro assay; living-cell model	0.2 U/mL	0.2–5 U/mL	NR	No	Experimental/cellular testing; not evaluated in clinical sepsis samples
Optical biosensor–FOLSPR	HMGB1; [69]	Anti-HMGB1 antibody	Fiber-optic localized surface plasmon resonance; anti-HMGB1 immobilized on a carboxymethyl-dextran-modified AuNP sensing surface	Standard HMGB1 solutions	43.4 pg/mL (1.7 pM)	10^−10^–10^−6^ g/mL	<10 min	No	Analytical/experimental evaluation; not validated in patients with sepsis
Electrochemical immunosensor	IL-1β + TNF-α; [70]	Capture antibodies + biotinylated detection antibodies (sandwich immunoassay)	Amperometric detection; functionalized DWCNT-modified dual screen-printed carbon electrodes with poly-HRP-streptavidin signal amplification	Spiked human serum; human saliva	IL-1β: 0.38 pg/mL; TNF-α: 0.85 pg/mL	IL-1β: 0.5–100 pg/mL; TNF-α: 1–200 pg/mL	2 h 30 min	Yes (2 analytes)	Biological-matrix validation (spiked human serum and human saliva); not evaluated in a clinical sepsis cohort
Nanoparticle-based biosensor	IL-6; [71]	Capture and detection anti-IL-6 antibodies	Plasmonic/colorimetric paper-based detection; AuNP nanoprobes with smartphone densitometric readout	Spiked human serum; spiked unprocessed whole blood	0.1 pg/mL	0.001–10 pg/mL†	<17 min	No	Analytical evaluation in spiked human serum and whole blood; not validated in a clinical sepsis cohort
Microfluidic/lab-on-chip biosensor	IL-6, TNF-α, CRP; [72]	Target-specific IL-6, TNF-α, and CRP aptamers	Label-free RIFTS; porous-silicon optical transducers integrated into a three-zone microfluidic chip	Experimental biomarker solutions	NR	NR	NR	Yes (3 analytes)	Experimental proof-of-concept; <7% signal variation between individual and multiplex measurements; no significant cross-reactivity; not clinically validated
Multiplex point-of-care platform	IL-6 + IL-8 + IL-10 + TRAIL + IP-10; [66]	Target-specific capture probes	Non-faradaic electrochemical impedance spectroscopy; nanofilm semiconductor/metal electrode array (DETecT Sepsis/READ)	Undiluted human plasma (<40 μL)	~1 pg/mL	Biomarker-dependent	~5 min	Yes (5 analytes)	Clinical-sample evaluation (n = 40); Pearson’s *r* > 0.90 versus reference methods

## Data Availability

No new data were created or analyzed in this study. Data sharing is not applicable to this article.

## Abbreviations

The following abbreviations are used in this manuscript:

**Abbreviation**	**Full Term**
PD-1	Programmed cell death protein-1
PD-L1	Programmed death ligand-1
TNF	Tumor necrosis factor
IL	Interleukin
DNA	Deoxyribonucleic acid
TNFR1	Tumor necrosis factor receptor 1
TRAIL	Tumor necrosis factor-related apoptosis-inducing ligand
NLRP3	NLR family pyrin domain containing 3
NLR	NOD-like receptor
NOD	Nucleotide-binding oligomerization domain
ASC	Apoptosis-associated speck-like protein containing a caspase recruitment domain
GSDMD	Gasdermin D
RIPK	Receptor-Interacting Serine/Threonine-Protein Kinase
TRADD	Tumor necrosis factor receptor type 1-associated death domain protein
FADD	Fas-associated death domain
MLKL	Mixed lineage kinase domain-like protein
PS	Phosphatidylserine
TUNEL	Terminal deoxynucleotidyl transferase dUTP nick-end labeling
FasL	Fas ligand
POC	Point-of-care
ALT	Alanine aminotransferase
AST	Aspartate aminotransferase
NT-proBNP	N-terminal pro-B-type natriuretic peptide
SOFA	Sequential Organ Failure Assessment
PLR	platelet-to-lymphocyte ratio
NLMR	neutrophil-to-lymphocyte-to-monocyte ratio
NK	Natural killer lymphocytes
PBMC	Peripheral blood mononuclear cell
APACHE	Acute Physiology and Chronic Health Evaluation
hs-cTnI	High-sensitivity cardiac troponin I
BUN	Blood urea nitrogen
CRP	C-reactive protein
qSOFA	quick SOFA
AuNP	Gold nanoparticle
DWCNT	Double-walled carbon nanotube
FOLSPR	Fiber-optic localized surface plasmon resonance
HMGB1	High-mobility group box 1
HRP	Horseradish peroxidase
IP-10	Interferon-γ-inducible protein 10
NR	Not reported
RIFTS	Reflectometric interference Fourier transform spectroscopy
SPR	Surface plasmon resonance
rGO	Reduced graphene oxide
AI	Artificial intelligence
